# An increase in IL-1β concentrations in embryo culture-conditioned media obtained by in vitro fertilization on day 3 is related to successful implantation

**DOI:** 10.1007/s10815-015-0573-4

**Published:** 2015-09-26

**Authors:** Karina Sequeira, Aurora Espejel-Núñez, Eva Vega-Hernández, Anayansi Molina-Hernández, Patricia Grether-González

**Affiliations:** Department of Reproduction and Infertility, National Institute of Perinatology, Montes Urales 800 Lomas Virreyes, CP: 11000 México DF, México; Department of Biochemistry and Molecular Biology, National Institute of Perinatology, México DF, México; Department of Cell Biology, National Institute of Perinatology, México DF, México; Department of Human Genetics and Genomics, National Institute of Perinatology, México DF, México

**Keywords:** Embryo, Implantation, Interleukin-1β, In vitro fertilization

## Abstract

**Purpose:**

This study aimed to evaluate interleukin (IL)-1β concentrations in maternal serum and in embryo-cultured conditioned media and to correlate these findings with success of implantation.

**Methods:**

A total of 70 infertile women who underwent in vitro fertilization treatment were studied. IL-1β concentrations were quantified in maternal serum and in embryo culture-conditioned media on days 1 and 3. The findings were compared between those who achieved pregnancy and those who did not.

**Results:**

No significant differences were found in IL-1β serum concentrations between the groups. IL-1β was not detected in day 1 culture-conditioned medium. On day 3, IL-1β was quantified in 27 patients, and IL-1β concentrations were significantly higher in women who achieved pregnancy than in those who did not (*p* < 0.001).

**Conclusions:**

High IL-1β concentrations in day 3 culture-conditioned medium in patients who achieve pregnancy after in vitro fertilization treatment indicate a possible role of embryonic IL-1β in the implantation process.

## Introduction

The success of growth and implantation of blastocysts is a complex event that involves maternal and embryonic signals. The interleukin-1 (IL-1) system comprises the following: two agonists, IL-1α and IL-1β, an antagonist IL-1RA, and two receptors, interleukin-1 receptor type I (IL-1R tI) and interleukin-1 receptor type II (IL-1R tII) [1, 2]. This system is closely associated with implantation. In humans, IL-1R tI has been detected in the uterine endometrium from day 23 of the menstrual cycle and IL-1β mRNA is increased from the proliferative to the secretory phase [3, 4].

IL-1β triggers the production of IL-8 by the endometrium, and this increases migration and survival of trophoblastic cells [5]. IL-1β increases secretion of prostaglandin E2 and leukemia inhibitory factor, as well as the expression of integrin α_ν_β_3_. In addition, the presence of IL-1β has been demonstrated in the villous cytotrophoblast, the syncytiotrophoblast, and the endometrial glands of the maternal decidua [6].

A reduction in integrin α_ν_β_3_ expression has been observed in luteal-phase deficiency, infertility of unknown cause, and in the hydrosalpinges. Interestingly, expression of this integrin is regulated by IL-1 that is secreted by embryos [7]. In the mouse, blockage of IL-1R tI with IL-1RA can in turn block implantation [8].

After the process of in vitro fertilization (IVF), implantation is positively correlated with high serum IL-1α and IL-1β concentrations. The implantation rates of patients in assisted fecundation cycles are higher in women who have greater IL-1β concentrations in blood on the day of human chorionic gonadotropin administration [9].

Barañao and colleagues [10] showed that IL-1β quantification in embryo culture-conditioned medium at 24 h of fertilization predicts the probability of pregnancy. In contrast, Seifer et al. [11] reported not having detected IL-1β in two- to six-cell human embryo supernatants. Another study showed that IL-1β mRNA expression was undetectable at the two-cell stage and was surprisingly low in the morula and blastocyst in mouse embryos [12]. De los Santos [13] demonstrated the presence of the whole IL-1 system in the embryo by means of immunohistochemistry. However, these authors were only able to find IL-1β secretion in the supernatant of embryos when these were co-cultured with epithelial-endometrial cells during all developmental stages.

Currently, the significance of serum IL-1β concentrations during the IVF cycle has not yet been completely clarified. Additionally, the presence of IL-1β in culture-conditioned medium of embryos under development and its possible relationship with the mechanism of implantation are unclear. Therefore, this study aimed to evaluate the association of serum IL-1β concentrations, as well as those in embryo culture medium, with success of implantation.

## Materials and methods

A prospective cohort study was carried out at the National Institute of Perinatology between April 2011 and July 2012. The study was approved by the ethics and research committees. Women younger than 40 years of age, about to undergo IVF treatment, were invited to participate. Written informed consent was obtained from all of the patients who were enrolled in the study.

We collected two 3-mL samples of peripheral blood. The first sample was collected on day 1 of the cycle when the patient’s basal hormone values were quantified. The second sample was collected on the day that two follicles >18 mm were observed, immediately prior to the injection of chorionic gonadotropin (last day of hormone stimulation).

Controlled ovarian hyperstimulation was initiated on day 2 of the menstrual cycle, using two alternative schemes: (1) recombinant human follicle-stimulating hormone (Gonal-F; Merck Serono, Germany) or (2) recombinant human follicle-stimulating hormone and urinary menotropins (Merapur; Ferring Pharmaceuticals, Switzerland). The ovarian stimulation protocol was selected by each treating physician as follows: (1) a long standard protocol with a gonadotropin-releasing agonist (Lucrin; Abbott, USA) in only six patients or (2) a flexible antagonist protocol (Cetrotide; Merck Serono, Germany) in 58 patients. No patients were transferred when estradiol levels were greater than 3500 pg/ml or progesterone levels were greater than 0.15 ng/ml.

For IL-1β quantification in serum, peripheral blood was centrifuged at 3000 rpm for 5 min. The serum was collected and frozen at −70 °C for later processing.

The oocytes were analyzed in the Assisted Reproduction Laboratory to define their morphological state and were incubated for approximately 4 h prior to fertilization. Standard IVF and intracytoplasmic sperm injection were performed*.* After fertilization, the zygotes were cultured in G-1TM/G-1TM PLuS medium (Vitrolife, San Diego CA, USA). The culture media were recovered at 24 and 72 h after fertilization. On day 3 prior to embryo transfer, we carried out morphological evaluation of the embryos using the Lucinda Veeck classification [14]. The embryos were categorized based on morphological evaluation into three groups: only top (qualities 1, 2 according to Lucinda Veeck classification), top and non-top (qualities 1, 2, 3, 4), and only non-top (qualities 3, 4).

Each embryo was cultured separately, but samples were the result of a pooled medium culture of two or three embryos. Culture media were mixed because we needed a minimum of 15 μL for testing. Therefore, those patients who achieved a unique embryo and patients who failed to be transferred were excluded (*n* = 6). We included only patients with two or three transferred embryos. These samples were immediately frozen at −70 °C for later processing.

IL-1β concentrations were quantified by the enzyme-linked immunosorbent assay technique (cat. DY401; R&D Systems, USA) using 100 μL of serum or the total volume of the embryo medium culture mixture. A standard curve of 1.5–50 pg/mL with a sensitivity limit of 0.1 pg/mL was used to calculate IL-1β concentrations. Inter- and intra-assay coefficients of variation were <10 %. An absorbance reading was taken at 450 nm (Synergy™ HT; BioTek Instruments, Winooski, VT, USA).

Pregnancy or successful implantation was considered in patients who were positive for beta human chorionic gonadotropin (1 mIU/ml) 14 days post-transfer of embryos.

The results are expressed as mean ± standard error of the mean. Differences between groups were compared by the Mann–Whitney *U* test. Significant differences were considered as *p* < 0.05.

## Results

### Patients

The total number of patients studied was 64. All of the patients were Mexicans with a mean age of 34 ± 3 years. The threshold estradiol level on the day of human chorionic gonadotropin administration was 1582 ± 550 pg/ml. No correlation between the quantification of IL-1β and serum estradiol (*p* = 0.39) was found. The baseline characteristics of the patients are shown in Table 1. The group of women who achieved pregnancy was compared with the group of women who failed to do so. There were no significant differences in baseline characteristics between the two groups.

**Table 1:** Baseline characteristics of patients included in the study

	Pregnancy *n* = 29	No pregnancy *n* = 35	*U*	*Z*	*p*
Age (years): median (min-max)	34 (22–28)	34 (27–39)	493	−0.91	0.42
BMI (kg/m^2^): median (min-max)	24.6 (17–32)	24.3 (17–29)	484	−0.31	0.37
Years of infertility: median (min-max)	5 (1–8)	7 (3–8)	427	−1.08	0.13
Follicles >18 mm: median (min-max)	7 (3–14)	5 (3–18)	466	−0.56	0.28
Estradiol (pg/ml) the day of hCG median (min-max)	1386 (1109–2548)	1633 (820–3394)	495	−0.16	0.43
Number or embryos transferred:			485	−0.39	0.34
2: *n* (%)	7 (24.1)	10 (28.6)			
3: *n* (%)	22 (75.9)	25 (71.4)			
Quality of transfer embryos:			395	−1.81	0.79
Only top (quality 1, 2): *n* (%)	22 (75.9)	20 (57.1)			
Top and non-top (quality 1, 2, 3, 4): *n* (%)	7 (24.1)	10 (28.6)			
Only non-top (quality 3, 4): *n* (%)	0 (0.0)	5 (14.3)			

Mann–Whitney *U* test. *BMI* body mass index, *hCG* human chorionic gonadotropin. No significant differences were found between the two groups

Standard IVF was performed in 44 (68.8 %) patients and intracytoplasmic sperm injection in 20 (31.3 %). The study participants were divided into two groups: 29 patients had a successful implantation or pregnancy and 35 patients did not achieve pregnancy. In the group of patients who failed to get pregnant, a total of 80 embryos were transferred, while in the group that achieved pregnancy, 95 embryos were transferred.

### Quantification of IL-1β concentrations in maternal serum

Serum quantification of IL-1β at initiation of treatment on day 1 was 0 pg/mL in 96.8 % (*n* = 62) of patients. In serum samples from the last day of controlled ovarian hyperstimulation, 47 patients (73 %) had detectable IL-1β concentrations, with a mean concentration of 39.5 ± 10.3 pg/mL. No difference in IL-1β concentrations was found between patients who achieved pregnancy and those that failed to do so (52.7 ± 22.0 vs. 28.7 ± 5.2 pg/mL; *p* = 0.29).

### Quantification of IL-1β concentrations in embryo culture-conditioned medium

On day 1 post-fertilization, we did not detect IL-1β concentrations (0 pg/mL in 64 patients). The percentage of detection of IL-1β concentrations on day 3 of embryo development was 42 % (27 patients), with a mean concentration of 4.1 ± 0.82 pg/mL.

**Fig. 1:**
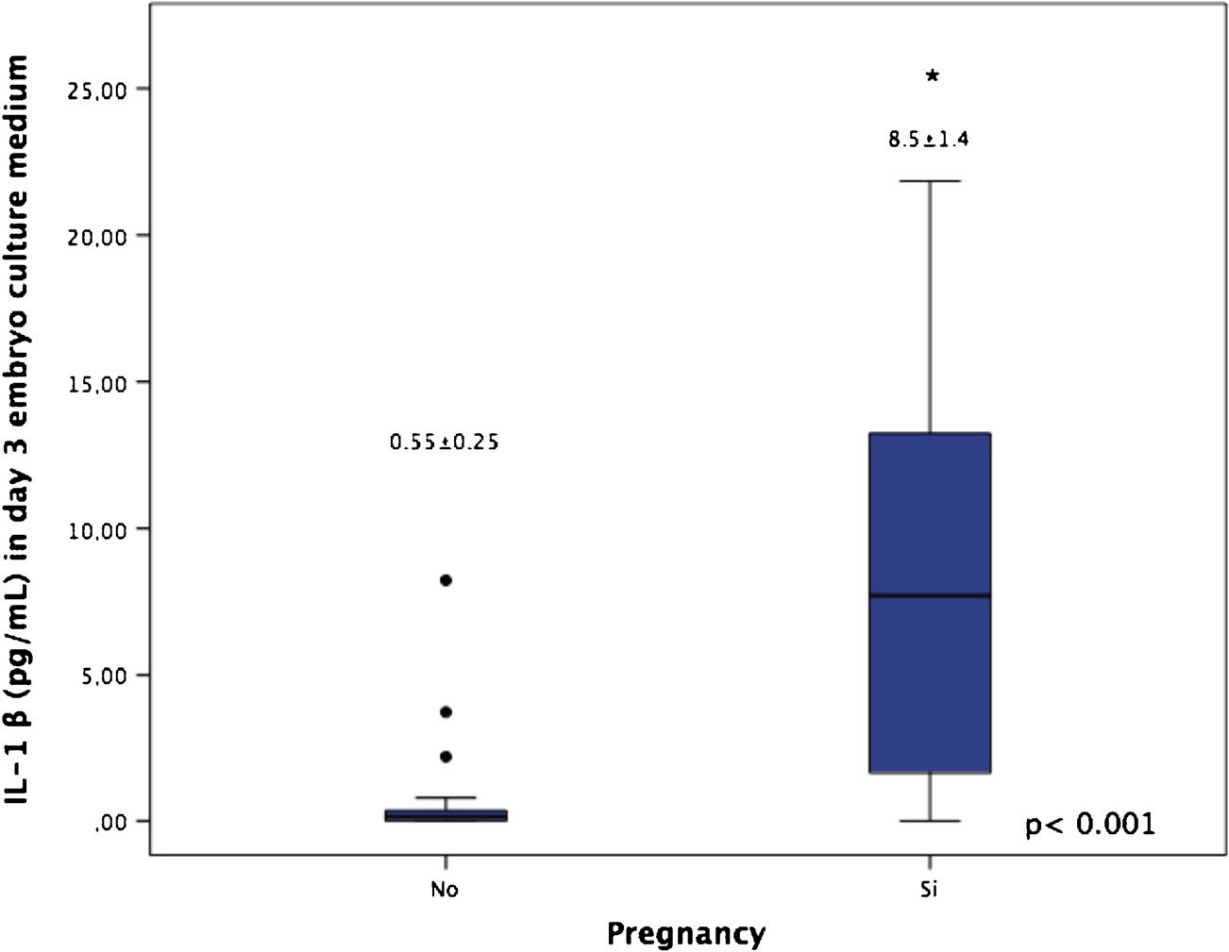
Box and whisker plot shows IL-1β concentrations in embryo culture medium at day 3 and their relation to successful implantation. No pregnancy, *n* = 35; pregnancy, *n* = 29. The results are expressed as mean ± standard error of the mean. *Boxes* represent the median, and the 25th and 75th percentiles. *Asterisk* indicates a significant difference (*p* < 0.001) between the two groups of IVF patients

We found that IL-1β concentrations of embryo supernatants of patients who achieved pregnancy were significantly higher than those in patients who did not achieve pregnancy (8.5 ± 1.4 vs. 0.55 ± 0.25 pg/mL; *p* < 0.001; Fig. 1).

## Discussion

One of the major issues in IVF is why patients who are submitted to repeated cycles of IVF with transfer of apparently good-quality embryos do not achieve pregnancy. This inexplicable failure in implantation is the basis of the current study.

Our study failed to detect any significant differences in serum IL-1β concentrations after stimulation of hormones. Karagouni et al. [9] detected serum IL-1β concentrations in 58 % of 33 patients with post-hCG concentrations of 68.5 ± 24.6 pg/mL in pregnant women and 20.5 ± 13.4 pg/mL in women without pregnancy. The reason for this difference between studies could be because hCG stimulates IL-1β secretion. In the present study, IL-1β was quantified after hormonal stimulation and prior to hCG administration to determine if elevation of IL-1β could have been related to the administration of FSH/MHG alone. Our findings indicated that controlled ovarian hyperstimulation induces systemic production of IL-1β. Therefore, IL-1β may have a regulatory effect on previous administration of hCG.

Our study showed IL-1β concentrations in embryo medium culture on day 3 of development and their correlation with pregnancy. Previous studies have suggested that embryos are not capable of secreting IL-1β if they are not co-cultured with endothelial cells. However, we were able to detect IL-1β in embryo medium culture at 3 days of development (eight cells) [13].

Notably, even though we did not achieve detection of IL-1β in a high proportion of patients*,* those who were positive showed an association between IL-1β concentrations and pregnancy. This finding suggested that IL-1β production could play an important role in the implantation process.

Barañao et al. [10] reported IL-1β concentrations of 49 ± 7 pg/mL in embryo culture supernatant at 24 h, but we did not find a detectable amount of IL-1β on day 1. This difference between studies may be due to the fact that we cultured single embryos per well and Barañao et al. [10] cultured a mean number of 5 ± 1 embryos per well. However, our finding is in agreement with that reported by Seifer et al. [11] who did not achieve quantification of IL-1β of two- to six-cell human embryo supernatants (on days 1 and 2). Our finding is also consistent with that reported in mice by Kruessel and colleagues, who did not find mRNA expression of IL-1β in the two-cell stage [12].

Four of the 37 patients who were negative for IL-1β in day 3 supernatants achieved pregnancy. The use of pooled media limited the level of the analysis because we quantified mean IL-1β production of two or three embryos and not individual IL-1β production. However, we consider that IL-1β synthesis could have started later in these embryos without impairing the implantation process.

While the IL-1 system is known to be associated with implantation, various questions with respect to its regulation still remain. Knowledge of the normal expression patterns of this cytokine can aid in predicting success in implantation, in selecting the embryos to be transferred in IVF programs, and in increasing the success of pregnancy.
